# The association between denture use and cardiovascular diseases. The United States National Health and Nutrition Examination Survey 2009–2018

**DOI:** 10.3389/fcvm.2022.1000478

**Published:** 2023-01-10

**Authors:** Xiaopeng Liang, Oscar Hou In Chou, Bernard M. Y. Cheung

**Affiliations:** ^1^Division of Clinical Pharmacology and Therapeutics, Department of Medicine, School of Clinical Medicine, The University of Hong Kong, Pokfulam, Hong Kong SAR, China; ^2^State Key Laboratory of Pharmaceutical Biotechnology, The University of Hong Kong, Pokfulam, Hong Kong SAR, China; ^3^Institute of Cardiovascular Science and Medicine, The University of Hong Kong, Pokfulam, Hong Kong SAR, China

**Keywords:** denture, cardiovascular diseases, coronary heart diseases, heart failure, National Health and Nutrition Examination Survey (NHANES)

## Abstract

**Introduction:**

Poor dental health is associated with cardiovascular diseases (CVD). However, the relationship between CVD and denture use is currently unknown. This study aimed to investigate whether denture use is associated with CVD among American adults.

**Methods:**

10,246 non-pregnant subjects aged 30–59 years from five cycles (2009–2018) of the United States National Health and Nutrition Examination Survey (NHANES) were included in this study. Participants who were observed by a dental examiner wearing denture/partial denture/plates were defined as denture users. CVD was defined as self-reported coronary heart disease, myocardial infarction, angina pectoris, stroke, and congestive heart failure. The association between denture use and CVD was analyzed using logistic regression with adjustment for potential cofounders.

**Results:**

4.4% (95% CI, 3.9–5.0) participants had CVD, and 3.5% (95% CI, 2.8–4.5) participants were denture users. Denture use was associated with CVD [OR = 4.26, 95% CI (2.90–6.28), *P* < 0.01], which remained significant [adjusted OR = 1.82, 95% CI (1.15–2.88), *P* < 0.01] after adjustments for sociodemographic characteristics, smoking, alcohol use, drug addiction, body mass index (BMI), and abnormal medical conditions including gum problem, hypertension, diabetes, and hyperlipidemia. Women with dentures had significantly higher odds of CVD [adjusted OR = 2.13, 95% CI (1.10–4.11), *P* = 0.025].

**Conclusion:**

In this nationally representative survey, denture use was associated with CVD. Denture use may be an unconventional risk factor for assessing CVD risks, especially in women. Future studies are required to investigate whether CVD and denture use is causally related.

## Introduction

Cardiovascular diseases (CVD), such as coronary heart disease, stroke, and congestive heart failure, are posing increasing burdens to the healthcare systems (1). Controlling conventional risk factors, such as blood pressure, total cholesterol, high-density lipoprotein cholesterol, smoking, and glucose intolerance, are essential in preventing CVD (2, 3). However, as individuals without conventional risk factors may still develop CVD, there is an increasing awareness that there are unrecognized potential risk factors which may also contribute to developing CVD. It was estimated that 10–20% of CVD can be explained by unconventional risk factors (4).

Poor oral health has been proposed as an unconventional risk factor for heart disease for many years. Previous studies have demonstrated that poor dental health has been related to an increased risk of all-cause mortality and mortality due to CVD and respiratory diseases (5). Vice versa, CVD and related treatments also affect dental health. Nevertheless, the results on the relation between oral diseases and CVD are so far inconsistent (6, 7). A recent meta-analysis demonstrated that dental problem was not associated with cardiovascular mortality (6).

Denture status is a strong predictor of oral health-related quality of life (8). However, denture problems, particularly denture-related stomatitis, are correlated with endothelial dysfunction in elderly patients (9). Endothelial dysfunction is linked to CVD by several mechanisms, including altered endothelial anticoagulant and anti-inflammatory capabilities, poor modulation of vascular growth, and uncontrolled vascular remodeling (10). While endothelial dysfunction may explain the potential link between dentures and CVD, the association between dentures and CVD has not been explored.

The present study aimed to investigate the association between denture use and CVD using data from the US National Health and Nutrition Examination Survey (NHANES) 2009–2018.

## Subjects and methods

### Study subjects

**FIGURE 1:**
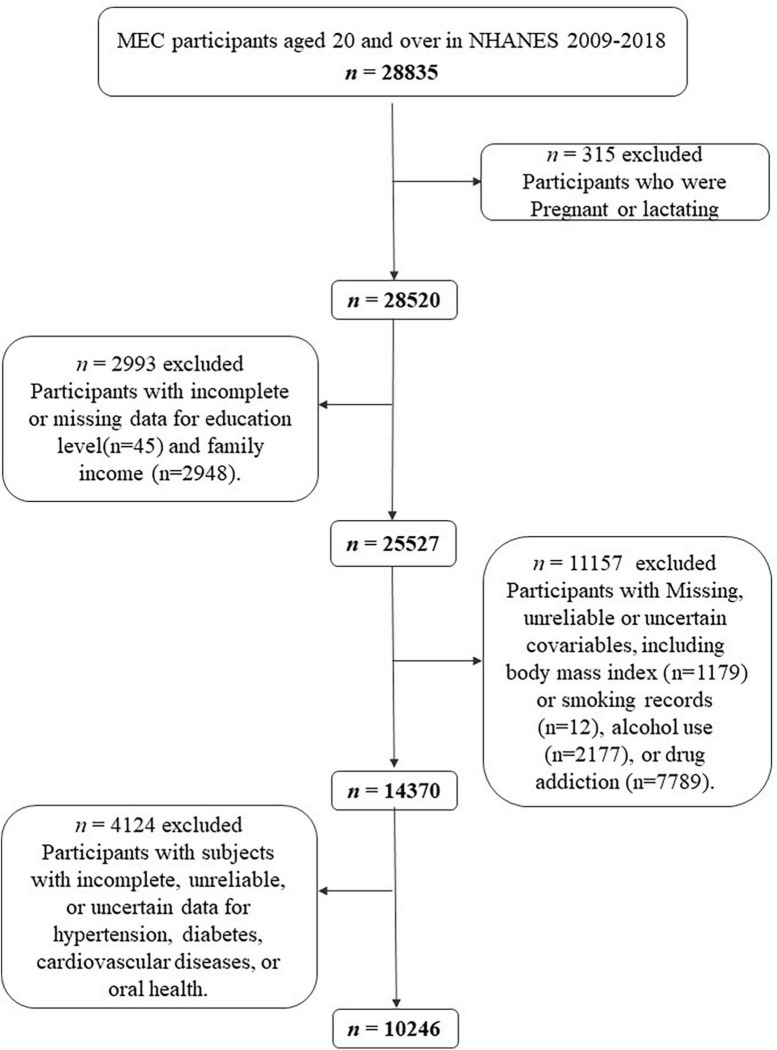
Flow chart of the screening process for the selection of eligible participants in National Health and Nutrition Examination Survey (NHANES) 2009–2018.

The National Center for Health Statistics of the US Centers for Disease Control and Prevention conducted NHANES 2009–2018. It is a cross-sectional survey of the civilian, non-institutionalized American population nationally representative samples (11). Data is released every 2 years, and the official website^1^ provides extensive descriptions of measuring processes and protocols (11). All participants provided informed consent, and the study was approved by the Institutional Review Board of the Centers for Disease Control and Prevention. In NHANES 2009–2018, 28,835 subjects were interviewed and examined in the mobile examination center. The demographics, examination results (blood pressure, body measures, oral health), medical condition questionnaire, questionnaire data (smoking, drug use, alcohol intake), and laboratory data (glycated hemoglobin, plasma fasting glucose) of the participants were extracted. A total of 315 pregnant or lactating participants were excluded. Participants with absence or incomplete data for education levels (*n* = 45), and family income-to-poverty ratio (*n* = 2,948) were also excluded. Missing, unreliable or uncertain covariables, including body mass index (BMI) (*n* = 1,179) or smoking records (*n* = 12), alcohol use (*n* = 2,177), or drug addiction (*n* = 7,789) were excluded. We also excluded 4,124 subjects with incomplete, unreliable, or uncertain data for hypertension, diabetes, CVD, or oral health (Figure 1).

### Definition

#### Sociodemographic variables

Participants were stratified into three age groups: 30–39, 40–49, and 50–59 years old. Race/ethnicity was defined as non-Hispanic whites, non-Hispanic blacks, Mexican Americans, and others. Three categories of educational attainment were used: high school or less, some college, and college graduates or above. Family income was divided into three categories based on the income-to-poverty ratio: less than 130%, 131–338%, and more than 339%.

#### Risk factors and disease

A BMI of less than 18.5 was considered underweight, BMI falling between 18.6–24.9 was regarded normal, over 25 was considered overweight, and over 30 was considered obese (12). Participants were regarded as “Never smoker” if they smoked less than 100 cigarettes in their lifetime; participants who identified as “ever smokers” had smoked over 100 cigarettes in their lifetime but had since quit. “Current light smokers” were participants who smoke less than 10 cigarettes per day now, and “current heavy smokers” were participants who smoke over 10 cigarettes per day. Alcohol intake was defined as having at least 12 drinks of any type of alcoholic beverage in any 1 year. A participant who self-reported taking cocaine, methamphetamines, or heroin more than two to five times in their lifetime was considered to have a drug addiction, which is defined as recurrent drug use. Diabetes was characterized as either a self-reported physician diagnosis of diabetes or increased values of fasting glucose [7.0 mmol/L (126 mg/dl)], glycated hemoglobin (HbA1c) [6.5%], or non-fasting glucose [11.1 mmol/L (200 mg/dl)]. Elevated levels of total cholesterol (≥240 mg/dl), triglycerides (≥200 mg/dl), or low-density lipoprotein (≥160 mg/dl) are referred to as hyperlipidemia (13). According to the American Heart Association/American College of Cardiology (AHA/ACC) 2017 guideline, participants were deemed to have hypertension if their systolic or diastolic blood pressure was greater than 130 or 80 mmHg, respectively (14). The definition of CVD (coronary heart disease, angina, myocardial infarction, congestive heart failure, or stroke) was obtained from the self-reported questionnaire. Participants who were observed by a dental examiner wearing denture/partial denture/plates were defined as denture users.

### Statistical analysis

Examination complex sample weights were adopted in all analyses due to unequal probabilities of selection, non-response bias, and oversampling of non-Hispanic blacks by using primary sampling units and strata. Data are presented as a mean (±standard error) or a percent (95% confidence interval). Multiple logistic regression models were applied to assess the trends over time. The association of dentures with CVD was assessed by multiple logistic regression. The *P* value for interaction was estimated by including each multiplicative interaction term in the linear regression models after adjusting for the main effects of all covariates. The odds ratio (OR) was adjusted to account for the presence of risk factor. All two-tailed significance tests have a *P* value of less than 0.05 to qualify as significant. STATA was used to conduct the statistical analysis (version 15.1).

## Results

### Characteristics of study participants

A total of 10,246 non-pregnant adults aged 30 years old were included in the final analysis. Each participant represented approximately 50,000 individuals in the United States. This sample size represented 97, 661, 993 civilians, a non-institutionalized U.S. population. The characteristics of participants by NHANES cycles and the complex sample weighted percentages of each character are summarized in Supplementary Table 1. Overall, 3.5% of (95% CI, 2.8–4.5%) subjects used dentures, and 4.4% (95% CI, 3.9–5.0%) of subjects had a history of CVD.

The characteristics of 10,246 participants stratified by denture status are shown in Table 1. The mean age of denture use was 50.14 years (95% CI 49.80–51.20). Over half of denture subjects were aged between 50 and 59 [55.8% (95% CI, 49.0–62.4)]. 53.4% (95% CI, 46.4–60.3) of denture users were men. Most frequently, those who were wearing dentures were participants with high school or below education [64.3% (95% CI, 58.5–69.7)], lower income (PIR < 138%) [46.5% (95% CI, 39.8–53.3)], obese [48.4% (95% CI, 43.0–53.8)], and current smokers [50.8% (95% CI, 43.6–58.0)]. In comparison to participants without dentures, denture users were more likely to have diabetes and hypertension (both *P* < 0.01). The crude rate of CVD amongst denture users was significantly higher than those non-denture users [15.2% (95% CI, 11.3–20.3) vs. 4.0% (95% 3.5–4.7)].

**TABLE 1:** Baseline characteristics of presence of denture patients versus the absence of denture patients.

Characteristics	Absence of denture problem (*n* = 9,762)		Presence of denture problem (*n* = 484)		*P* value
	**Unweighted *N***	**Weighted % (95% CI)**	**Unweighted *N***	**Weighted % (95% CI)**	
**Age group**					
30–39 years	3,349	32.4 (30.9–33.9)	69	14.3 (10.2–19.9)	<0.01
40–49 years	3,310	33.6 (32.3–34.9)	146	29.8 (24.8–35.5)	0.032
50–59 years	3,103	34 (32.5–35.7)	269	55.8 (49–62.4)	<0.01
**Gender**					
Men	4,853	50.6 (49.5–51.7)	252	53.4 (46.4–60.3)	0.435
Women	4,909	49.4 (48.3–50.5)	232	46.6 (39.7–53.6)	0.435
**Race/Ethnicity**					
Non-Hispanic white	3,919	66.5 (63.1–69.7)	203	59.3 (50.7–67.4)	0.173
Non-Hispanic black	1,992	10.7 (9.2–12.3)	164	21.1 (15.5–28)	<0.01
Mexican Americans	1,461	8.8 (7.2–10.8)	42	6.7 (4.3–10.1)	0.423
Others	2,390	14 (12.6–15.6)	75	12.9 (9.3–17.7)	0.387
**Education level**					
High school below	3,898	32.9 (30.5–35.3)	308	64.3 (58.5–69.7)	<0.01
Some college	2,979	31.4 (30.1–32.7)	150	30.1 (25.8–34.8)	0.238
College graduate or above	2,885	35.7 (33–38.6)	26	5.6 (3.4–8.9)	<0.01
**Poverty to income ratio**					
≤130%	2,866	18.6 (16.9–20.4)	269	46.5 (39.8–53.3)	<0.01
131–338%	3,275	30.5 (28.7–32.3)	165	39.9 (33.6–46.7)	<0.01
≥339%	3,621	50.9 (48.4–53.4)	50	13.5 (9–19.8)	<0.01
**BMI (kg/m^2^)**					
Underweight (<18.5)	97	1 (0.8–1.3)	9	1.4 (0.6–3.1)	0.210
Normal (18.5–24.9)	2,452	25.3 (23.9–26.7)	117	25.9 (20–32.9)	0.462
Overweight (25.0–29.9)	3,174	33.4 (32.1–34.8)	129	24.3 (18.9–30.6)	<0.01
Obese (≥30)	4,039	40.3 (38.6–42)	229	48.4 (43–53.8)	<0.01
**Smoking status**					
Never smokers	5,591	56.7 (54.9–58.5)	164	32.9 (27.8–38.5)	<0.01
Former smokers	1,959	23 (21.6–24.5)	83	16.2 (12.2–21.3)	<0.01
Current smokers	2,212	20.3 (18.9–21.8)	237	50.8 (43.6–58)	<0.01
Alcohol intake	8,692	91.9 (90.7–93)	431	90.8 (87.9–93)	0.415
Drug addiction	5,020	59.1 (57.1–61)	307	64.3 (57.5–70.7)	0.125
Diabetes	1,339	11.5 (10.7–12.3)	100	20.4 (16.1–25.7)	<0.01
Hypertension	5,477	55.2 (53.4–57)	356	72.7 (67.9–77)	<0.01
Hyperlipidemia	1,826	19.2 (18–20.4)	97	20.7 (14.8–28.3)	0.654
Gum problem	2,550	21.4 (19.5–23.4)	306	62.4 (55.8–68.6)	<0.01
CVD	465	4 (3.5–4.7)	64	15.2 (11.3–20.3)	<0.01
CHD	118	1.1 (0.9–1.5)	15	4.1 (2.1–7.9)	<0.01
MI	177	1.6 (1.3–2)	22	5.4 (3.1–9.4)	<0.01
Angina pectoris	118	1.1 (0.9–1.4)	20	6.1 (3.2–11.3)	<0.01
CHF	120	0.9 (0.7–1.1)	23	6.1 (3.8–9.7)	<0.01
Stroke	166	1.3 (1.1–1.6)	25	5.9 (3.9–9)	<0.01

CVD, cardiovascular diseases; CHD, coronary heart diseases; MI, myocardial infarction; CHF, congestive heart failure.

The characteristics of all 10,246 participants stratified by CVD status are shown in Table 2. The mean age of CVD subjects was 49.69 (95% CI, 48.87–50.51) years. 59.2% (95% CI, 53.5–64.7) of CVD subjects were aged between 50 and 59 years old. Subjects in high school or below, lower-income, current smokers, and obese had more CVD (all *P* < 0.01). Participants with CVD also had a higher prevalence of alcohol intake, drug addiction, gum problems, diabetes, and hypertension than non-CVD participants (all *P* < 0.01). The use of dentures was also more common among participants with CVD [12.1% (95% CI, 8.6–16.7) vs. 3.1% (95% CI, 2.4–4.0)].

**TABLE 2:** Baseline characteristics of participants with or without CVD.

Characteristics	Absence of CVD (*n* = 9,717)		Presence of CVD (*n* = 529)		*P* value
	**Unweighted *N***	**Weighted % (95% CI)**	**Unweighted *N***	**Weighted % (95% CI)**	
**Age group**					
30–39 years	3,343	32.6 (31.2–34)	75	13.9 (10.8–17.8)	<0.01
40–49 years	3,308	33.7 (32.4–35.1)	148	26.9 (22.8–31.4)	<0.01
50–59 years	3,066	33.7 (32.1–35.3)	306	59.2 (53.5–64.7)	<0.01
**Gender**					
Men	4,828	50.4 (49.3–51.6)	277	55.8 (50.5–60.9)	0.056
Women	4,889	49.6 (48.4–50.7)	252	44.2 (39.1–49.5)	0.389
**Race/Ethnicity**					
Non-Hispanic white	3,875	66.2 (62.8–69.4)	247	65.8 (60.3–71)	0.273
Non-Hispanic black	2,013	10.8 (9.3–12.5)	143	15.7 (12.4–19.7)	0.003
Mexican Americans	1,453	8.9 (7.3–10.8)	50	6.2 (4.3–8.9)	0.045
Others	2,376	14.1 (12.6–15.7)	89	12.2 (9.5–15.7)	0.764
**Education level**					
High school below	3,935	33.3 (30.9–35.8)	271	48.7 (42.8–54.6)	<0.01
Some college	2,953	31.2 (29.8–32.5)	176	35.1 (29.6–41.1)	0.723
College graduate or above	2,829	35.5 (32.8–38.4)	82	16.2 (12.7–20.3)	<0.01
**Poverty to income ratio**					
≤130%	2,878	18.7 (17–20.5)	257	38 (32.9–43.4)	<0.01
131–338%	3,270	30.7 (28.9–32.5)	170	34.2 (29.4–39.3)	0.652
≥339%	3,569	50.6 (48.1–53.1)	102	27.8 (22.4–34)	<0.01
**BMI (kg/m^2^)**					
Underweight (<18.5)	98	1 (0.8–1.3)	8	1.8 (0.7–4.4)	0.749
Normal (18.5–24.9)	2,476	25.8 (24.4–27.2)	93	16 (12.2–20.7)	<0.01
Overweight (25.0–29.9)	3,171	33.4 (32.1–34.8)	132	25.7 (21.5–30.5)	<0.01
Obese (≥30)	3,972	39.8 (38.1–41.5)	296	56.5 (50.4–62.3)	<0.01
**Smoking status**					
Never smokers	5,573	57 (55.2–58.7)	182	32.6 (27.6–38)	<0.01
Former smokers	1,916	22.6 (21.2–24.1)	126	26.1 (20.8–32.3)	<0.01
Current smokers	2,228	20.4 (19.1–21.9)	221	41.3 (35.6–47.2)	<0.01
Alcohol intake	8,627	91.7 (90.5–92.8)	496	95 (92.2–96.8)	0.015
Drug addiction	4,990	58.9 (56.9–60.8)	337	67 (62.6–71)	<0.01
Diabetes	1,279	11 (10.3–11.8)	160	28.2 (23.6–33.2)	<0.01
Hypertension	5,397	54.9 (53.1–56.6)	436	77 (71.4–81.7)	<0.01
Hyperlipidemia	1,817	19.1 (17.9–20.4)	106	20.8 (16.9–25.4)	0.458
Gum problem	2,674	22.4 (20.6–24.4)	182	30.6 (26.1–35.4)	<0.01
Denture problem	420	3.1 (2.4–4)	64	12.1 (8.6–16.7)	<0.01

CVD, cardiovascular diseases.

### The association between dentures and CVD

In the competing risk analysis, denture use was significantly associated with CVD [OR = 4.26 95% CI (2.90–6.28), *P* = 0.01] (Table 3). Age, education levels, BMI, smoking, alcohol intake, drug addiction, diabetes, hypertension, and gum problems may have a potential effect on the association between dentures and CVD. The multivariate logistic regression analysis of the association between denture use and CVD before and after adjustments is shown in Table 4. Denture use was significantly associated with CVD after adjustments [OR = 1.82, (95% CI, 1.15–2.88)]. That is, denture use was independently associated with 82% higher odds of CVD. The association was particularly significant among women in the subgroup analysis (OR = 2.13, 95% CI: 1.10–4.11) (Table 5).

**TABLE 3:** Competing risk analysis of incident CVD.

CVD	Cause-specific odd ratio		
	**OR**	**95% CI**	***P*** **value**
Denture/Partial Denture/Plates	4.26	2.895–6.279	<0.01
Male	1.23	0.984–1.533	0.069
**Age (ref: 30–39)**			
40–49	1.81	1.333–2.451	<0.01
50–59	3.82	2.71–5.376	<0.01
**Race/Ethnicity (ref: others)**			
Non-Hispanic white	0.87	0.636–1.193	0.561
Non-Hispanic black	0.65	0.46–0.91	0.201
Mexican Americans	1.22	0.849–1.765	0.721
**Education (ref: <11th grade)**			
High school	1.22	0.926–1.614	0.093
College and above	2.86	2.108–3.883	<0.01
**Poverty to income ratio (ref: ≤130%)**			
131–338%	1.72	1.342–2.213	0.016
≥339%	3.28	2.346–4.581	<0.01
**BMI (ref: underweight)**			
Normal	2.80	0.919–8.507	0.317
Overweight	2.19	0.8–5.999	0.288
Obesity	1.23	0.47–3.229	0.152
**Smoking status (ref: never)**			
Former smokers	2.00	1.435–2.793	0.016
Current smoke	3.17	2.398–4.184	<0.01
Alcohol intake	1.75	1.085–2.833	0.022
Drug Addition	1.39	1.151–1.686	0.001
Diabetes	3.02	2.321–3.922	<0.001
Hypertension	2.62	1.945–3.53	<0.001
Hyperlipidemia	1.10	0.835–1.458	0.485
Gum problem	1.28	1.005–1.619	0.045

CVD, cardiovascular diseases.

**TABLE 4:** The association between denture and CVD.

	Crude OR		Model 1		Model 2		Model 3	
	**OR**	***p***	**OR**	***p***	**OR**	***p***	**OR**	***p***
CVD	4.26 (2.9–6.28)	<0.001	1.96 (1.28–2.99)	<0.001	1.82 (1.19–2.79)	0.006	1.82 (1.15–2.88)	0.011
CHD	3.71 (1.82–7.55)	<0.001	1.71 (0.83–3.5)	0.141	1.71 (0.85–3.43)	0.128	1.7 (0.78–3.71)	0.183
MI	3.44 (1.79–6.61)	<0.001	1.44 (0.75–2.79)	0.270	1.3 (0.67–2.54)	0.430	1.37 (0.66–2.85)	0.393
Angina	5.66 (2.77–11.53)	<0.001	2.82 (1.36–5.87)	<0.001	2.6 (1.26–5.37)	<0.001	2.86 (1.31–6.24)	0.009
CHF	7.42 (3.98–13.84)	<0.001	2.73 (1.34–5.58)	<0.001	2.73 (1.36–5.5)	0.005	2.75 (1.3–5.82)	0.009
Stroke	4.73 (2.75–8.13)	<0.001	2.06 (1.1–3.85)	0.025	1.84 (0.98–3.43)	0.056	1.84 (1.01–3.34)	0.046

CVD, cardiovascular diseases; CHD, coronary heart diseases; MI, myocardial infarction; CHF, congestive heart failure.

Model 1: adjusted for age, gender, race, education level, and income.

Model 2: adjusted for age, gender, race, education level, income, cigarette smoking, alcohol use, and drug addiction.

Model 3: adjusted for age, gender, race, education level, income, cigarette smoking, alcohol use, drug addiction, body mass index (BMI), and abnormal medical conditions including gum problem, hypertension, diabetes, and hyperlipidemia.

**TABLE 5:** Subgroup analysis of the association between denture and CVD.

	Men		Women	
	**OR**	***p***	**OR**	***p***
CVD	1.62 (0.82–3.19)	0.163	2.13 (1.1–4.11)	0.025
CHD	1.4 (0.45–4.33)	0.556	2.57 (0.6–10.99)	0.199
MI	1.68 (0.63–4.51)	0.297	0.98 (0.24–4.09)	0.982
Angina	2.09 (0.59–7.47)	0.253	4.14 (1.42–12.1)	<0.01
CHF	1.78 (0.42–7.67)	0.432	3.94 (1.62–9.59)	<0.01
Stroke	1.68 (0.79–3.58)	0.178	1.94 (0.91–4.14)	0.087

CVD, cardiovascular diseases; CHD, coronary heart diseases; MI, myocardial infarction; CHF, congestive heart failure.

All data adjusted for age, race, education level, income, cigarette smoking, alcohol use, drug addiction, body mass index (BMI), and abnormal medical conditions including gum problem, hypertension, diabetes, and hyperlipidemia.

## Discussion

Our study demonstrated a robust association between denture use and CVD after adjustments using 10 years of nationally representative data from United States NHANES 2009–2018. Our study indicated that dentures might be an unconventional risk factor for CVD.

Abundant studies showed that poor dental health was related to CVD (7, 15). A cohort study of a million participants with over 65,000 cardiovascular events found a moderate association between poor oral health and coronary heart disease (15). However, the majority of the previous studies were only investigating periodontal inflammation and gingival bleeding (16, 17). Dentures are used to maintain the continuity of the masticatory system and improve esthetic outcomes, which are essential in the restoration and rehabilitation of oral health (18, 19). The prevalence of denture-related lesions among denture users, including denture stomatitis, angular cheilitis, traumatic ulcers, denture irritation hyperplasia, flabby ridges, and oral carcinomas, varies from 50 to 75% (20, 21).

The denture-related stomatitis involves an inflammatory process upon oral mucosa contact with dentures. Indeed, over two-thirds of older individuals who wear dentures are affected, making it one of the most prevalent diseases in this population (21). Age, smoking, obesity, and diabetes are the common risk factors for both denture-related stomatitis and atherosclerotic vascular disease (22, 23). A previous study has shown that denture-related stomatitis was associated with a reduction in endothelial function (9). The severity of endothelial dysfunction is substantially correlated with the onset of coronary artery disease. It also predicts future cardiovascular events. Thus, dentures may directly increase the risk of cardiovascular disorders.

Besides, continuous and long-term usage of dentures may accelerate the formation of denture plaque on the surfaces of prostheses (22). A special plaque, made up of fat, cholesterol, calcium, and other substances in the blood, may accumulate as dental plaque and vascular plaque. As such, dental plaque could be a telltale sign of atherosclerosis, predicting the risk of CVD. Furthermore, fungi are also major colonizers of the denture-wearing oral cavity. Oral biofilms, which commonly contain three to four species of fungi, may lead to systemic infections (24). These breeding fungi and bacteria infections may also cause other oral problems such as cavities, gingivitis, and periodontal diseases, which affects the blood coagulation, lipid metabolism, thrombocytes, macrophages, and endothelial cells. As such, the denture plaque on the denture may also explain the increased cardiovascular risks.

Another plausible explanation is the different salivary and gut microbiome compositions between denture users and non-users. Previously, the salivary microbiome among 997 adults was analyzed using high-throughput sequencing of the V1–V3 region of the 16S rRNA gene. It demonstrated that *Streptococcus* and *Neisseria* were more prevalent micro-organisms among denture users. Moreover, the salivary microbiome among denture users was also less diverse (25). The salivary microbiome is a good reflection of the gut microbiome’s complexity and diversity (26). It is now well-known that the gut microbiome strongly predicts the development of CVD (27). Gut microbiota was shown to play a key role in various CVD, including atherosclerosis, hypertension, heart failure, atrial fibrillation, and myocardial fiber change (28, 29).

In the gender-stratified analysis, there was a strong association between dentures and CVD among women (Table 5). This difference may be attributed to hormonal changes (30). It may as well reflects the differences in the frequency of dental treatment between women and men (31). In the age-stratified analysis, the association remains significant for both men and women aged 40–49 years old. Advanced age is an established risk factor for both denture problems and CVD (32). A recent study demonstrated that denture use was associated with an increased risk of future ischemic events in patients with acute myocardial infarction, especially in those with age ≥ 75 years (33). However, in our study, the association did not increase with age, but rather, strongest among participants aged 40–49 years old. The difference could be explained by the different study populations and definitions of outcomes between the two studies, as well as the presence of confounding factors.

It is important to acknowledge the limitations of this study. Firstly, given the cross-sectional nature of the study and the presence of confounding factors such as lower socioeconomic status, obesity, and smoking, future studies are required to investigate whether CVD and denture use is causally related. While those factors were adjusted in the multivariate analysis, residual confounding might still be present. Besides, NHANES includes a self-reported questionnaire for medications, illnesses, and other health variables, which introduces recall and self-reported bias. Thirdly, the NHANES did not include information on disorders associated with dentures, such as denture stomatitis, traumatic ulcers, and oral cancers. Fourth, NHANES did not provide information on the duration of denture wearing, such that the effect of the duration on CVD was not investigated. Last but not least, the study did not take medication use into account. For instance, the effect of anticoagulant therapies on dental health was not discussed.

## Conclusion

Our analysis from a nationally representative survey demonstrated denture wearing was significantly associated with CVD, especially in women and participants between 40–49 years old. Denture usage may potentially be a risk factor for CVD. Future studies are required to investigate whether CVD and denture use are causally related.

## Data availability statement

The datasets presented in this study can be found in online repositories. The names of the repository/repositories and accession number(s) can be found in the article/Supplementary material.

## Ethics statement

The studies involving human participants were reviewed and approved by the Research Ethics Review Board at the National Center for Health Statistics in the US. The patients/participants provided their written informed consent to participate in this study.

## Author contributions

XL: conceptualization, methodology, data analysis, and drafting of the manuscript. OC: conceptualization and revision of the manuscript. BC: methodology, supervision, and revision of the manuscript. All authors contributed to the article and approved the submitted version.

## Footnotes

1
https://www.cdc.gov/nchs/nhanes/index.htm
