# Supplementary material for: The association between denture use and cardiovascular diseases. The United States National Health and Nutrition Examination Survey 2009–2018
Source: Front Cardiovasc Med. 2023 Jan 10;9:1000478. doi: 10.3389/fcvm.2022.1000478 (PMC9871755; doi:10.3389/fcvm.2022.1000478)
Supplement: Supplementary file 1 [file Data_Sheet_1.docx]

**Supplementary Table 1. Characteristics of participants included in this analysis in NHANES 2009-2018.**

| Characteristics | Total | 2009-2010 | 2011-2012 | 2013-2014 | 2015-2016 | 2017-2018 | P value |
| --- | --- | --- | --- | --- | --- | --- | --- |
| N | 10246 | 2103 | 1955 | 2219 | 2074 | 1895 |  |
| Age group |  |  |  |  |  |  | 0.838 |
| 30-39 years | 31.8(30.4-33.1) | 31.3(29.1-33.7) | 30.8(27.3-34.6) | 31.7(29.6-33.9) | 31.7(28.2-35.5) | 33(30-36.2) |  |
| 40-49 years | 33.4(32.1-34.8) | 35.4(33-37.9) | 34.1(31.3-37) | 33.2(30.7-35.7) | 33.4(30.3-36.7) | 31.4(28.3-34.7) |  |
| 50-59 years | 34.8(33.3-36.4) | 33.3(30.4-36.2) | 35.1(32-38.2) | 35.1(32.3-38) | 34.9(30.8-39.1) | 35.6(31.9-39.5) |  |
| Women | 49.3(48.2-50.4) | 47.9(45.7-50.1) | 49(45.6-52.4) | 49.4(47.6-51.1) | 50.7(48.6-52.8) | 49.4(46.9-52) | 0.507 |
| Ethnicity |  |  |  |  |  |  | 0.385 |
| Non-Hispanic White | 66.2(62.9-69.4) | 70.1(63.2-76.1) | 68.3(60.3-75.3) | 66.6(59.8-72.9) | 65.1(55.9-73.3) | 61.4(54.9-67.6) |  |
| Non-Hispanic Black | 11(9.5-12.7) | 11.1(9.4-13.1) | 11.2(7.5-16.4) | 11(8.5-14) | 10.9(7.1-16.4) | 11(8-14.9) |  |
| Mexican Americans | 8.8(7.2-10.7) | 8.2(4.7-14) | 7.5(4.8-11.8) | 9.3(6.1-14) | 9.2(5.6-14.7) | 9.4(6.7-13) |  |
| Others | 14(12.6-15.6) | 10.6(7.9-14.1) | 13(10.3-16.3) | 13(10.7-15.8) | 14.8(11.4-19) | 18.2(14.8-22.2) |  |
| Education level |  |  |  |  |  |  | 0.139 |
| High school below | 34(31.6-36.4) | 38.3(34.7-41.9) | 34.6(28.8-40.8) | 33.6(27.9-39.8) | 31.3(25.8-37.4) | 32.8(28.8-37.2) |  |
| Some College | 31.3(30-32.7) | 28.9(26.8-31.1) | 29.5(26.4-32.7) | 31.9(29.1-34.9) | 32.6(29.5-35.9) | 33.4(30.5-36.4) |  |
| College graduate or above | 34.7(32-37.4) | 32.8(29.6-36.2) | 36(30-42.4) | 34.5(28.5-41) | 36.1(29-43.8) | 33.8(28.2-39.8) |  |
| Poverty to income ratio |  |  |  |  |  |  | 0.685 |
| ≤130% | 19.6(17.8-21.4) | 17.9(15.6-20.4) | 21.3(17.5-25.5) | 22(16.3-29.1) | 18(14.8-21.5) | 18.6(17-20.3) |  |
| 131%-338% | 30.8(29.1-32.7) | 29.7(26.9-32.8) | 31.5(27.4-35.8) | 30.2(27.3-33.3) | 33.2(28.4-38.4) | 29.4(25.2-34) |  |
| ≥339% | 49.6(47.1-52.1) | 52.4(49.3-55.4) | 47.3(41-53.6) | 47.8(41.4-54.2) | 48.8(42.1-55.6) | 52(47.8-56.2) |  |
| BMI group |  |  |  |  |  |  | 0.040 |
| Underweight (<18.5) | 1.1(0.8-1.3) | 1.3(0.8-2.3) | 0.8(0.5-1.5) | 0.7(0.4-1.3) | 0.9(0.5-1.7) | 1.5(1-2.3) |  |
| Normal (18.5-24.9) | 25.3(24-26.7) | 26.2(23.4-29.2) | 26.5(24.4-28.8) | 24.9(22.4-27.6) | 25.2(21.5-29.4) | 23.9(21.1-27) |  |
| Overweight (25.0-29.9) | 33.1(31.8-34.4) | 35(32-38.1) | 35.4(32.8-38) | 33.7(31-36.6) | 32.2(29.6-34.9) | 29.4(26.2-32.9) |  |
| Obese (≥30) | 40.5(38.9-42.2) | 37.4(35.2-39.7) | 37.2(34.2-40.3) | 40.7(36.7-44.7) | 41.7(37.1-46.4) | 45.2(41.6-48.8) |  |
| Smoking status |  |  |  |  |  |  | 0.689 |
| Never smokers | 55.9(54.1-57.6) | 55.8(51.7-59.9) | 53.7(51.2-56.2) | 56.7(52.6-60.7) | 55.6(52.1-59) | 57.5(52.7-62.1) |  |
| Former smokers | 22.8(21.4-24.2) | 23(18.9-27.7) | 23.5(20.2-27.1) | 21(18.7-23.6) | 23.9(21.2-27) | 22.5(20-25.2) |  |
| Current smokers | 21.4(20-22.8) | 21.2(19-23.5) | 22.8(19.6-26.4) | 22.3(19.2-25.7) | 20.5(17.4-23.9) | 20.1(17.1-23.4) |  |
| Alcohol intake | 91.9(90.7-92.9) | 92.5(91.1-93.7) | 92.3(90.6-93.7) | 89.7(84.6-93.3) | 90.3(87.9-92.2) | 94.6(92.7-96.1) | 0.035 |
| Drug Addiction | 13.2(10.3-16.1) | 17.4(11.8-22.8) | 10.7(6.2-15.1) | 10.3(6.4-14.0) | 17.2(12.9-21.4) | 10.5(7.3-13.6) | 0.649 |
| Diabetes | 11.8(11-12.6) | 8.9(7.6-10.4) | 11.8(10.5-13.3) | 12.1(10.3-14.2) | 12.9(10.8-15.2) | 12.8(11.3-14.4) | 0.006 |
| Hypertension | 55.8(54.1-57.5) | 51.5(48.2-54.8) | 56.6(52.6-60.6) | 53.5(49.4-57.5) | 58.5(54.6-62.2) | 58.6(54.9-62.3) | 0.025 |
| Hyperlipidemia | 19.2(18.1-20.4) | 19.6(17.8-21.5) | 22(19-25.3) | 19.2(17.4-21.1) | 19.4(16.9-22.2) | 16(13.2-19.2) | 0.147 |
| Gum problem | 22.8(21-24.8) | 29.8(25.9-34.1) | 37.9(32.6-43.6) | 33.6(28.6-39) | 4.6(3.1-6.8) | 9.6(6.7-13.6) | <0.01 |
| Denture Problem | 3.5(2.8-4.5) | 1.3(0.8-2.3) | 7.4(5.1-10.6) | 5.3(3.4-8.1) | 1.1(0.5-2.4) | 2.4(1.1-5.1) | 0.07 |
| Cardiovascular diseases | 4.4(3.9-5) | 4.1(3.5-5) | 4.6(3.6-5.9) | 4.4(3.2-6) | 4.5(3.4-6) | 4.5(3.3-6.1) | 0.962 |
| CHD | 1.2(1-1.6) | 1.1(0.7-1.8) | 1.4(0.8-2.5) | 1.3(0.7-2.4) | 1(0.6-1.8) | 1.4(0.9-2) | 0.921 |
| Angina pectoris | 1.3(1.1-1.6) | 1(0.6-1.5) | 1.4(0.7-2.6) | 1.3(0.8-1.9) | 1.8(1.1-2.8) | 1.2(0.8-1.8) | 0.421 |
| MI | 1.8(1.5-2.1) | 1.9(1.3-2.7) | 1.6(1.1-2.3) | 1.6(1.1-2.5) | 1.9(1.4-2.8) | 1.8(1-3.2) | 0.917 |
| CHF | 1.1(0.8-1.3) | 0.7(0.4-1.2) | 1.3(0.8-2.2) | 1.4(0.9-2) | 0.8(0.5-1.2) | 1.1(0.6-1.8) | 0.146 |
| Stroke | 1.5(1.2-1.8) | 1.4(1-1.9) | 1.5(1-2.1) | 1.3(0.9-1.9) | 1.4(0.9-2.2) | 1.8(1.2-2.5) | 0.819 |

**Supplementary Table 2. Subgroup analysis of the association between denture and CVD**

|  | OR | p | p for interaction |
| --- | --- | --- | --- |
| Age |  |  | 0.092 |
| 30-39 years | 2.37(0.47-11.9) | 0.291 |  |
| 40-49 years | 2.93(1.44-5.96) | 0.004 |  |
| 50-59 years | 1.56(0.85-2.86) | 0.148 |  |
| Men |  |  | 0.051 |
| 30-39 years | 4.31(0.87-21.27) | 0.072 |  |
| 40-49 years | 3(1.14-7.87) | 0.026 |  |
| 50-59 years | 1.42(0.58-3.47) | 0.436 |  |
| Women |  |  | 0.071 |
| 30-39 years | 1.29(0.08-21.83) | 0.858 |  |
| 40-49 years | 2.95(1.12-7.74) | 0.029 |  |
| 50-59 years | 1.69(0.77-3.7) | 0.184 |  |
| Blood pressure |  |  | 0.231 |
| Normal BP | 3.85(1.83-8.1) | <0.01 |  |
| Elevated BP | 0.8(0.35-1.81) | 0.579 |  |
| Hypertension stage 1 | 1.66(0.58-4.74) | 0.343 |  |
| Hypertension stage 2 | 1.09(0.4-2.97) | 0.863 |  |
| LDL-cholesterol (mmol/L) |  |  | 0.072 |
| Quartile 1 (<2.48 in men and <2.35 in women) | 2.62(1-6.83) | 0.049 |  |
| Quartile 2 (2.48–2.95 in men and 2.35–2.95 in women) | 1.93(0.68-5.51) | 0.215 |  |
| Quartile 3 (2.96–3.60 in men and 2.95–3.54 in women) | 0.49(0.1-2.33) | 0.361 |  |
| Quartile 4 (≥3.61 in men and ≥3.55 in women) | 0.87(0.25-3.06) | 0.829 |  |
| Triglyceride (mmol/L) |  |  | 0.051 |
| Quartile 1 (<0.85 in men and <0.70 in women) | 2.36(0.39-14.22) | 0.345 |  |
| Quartile 2 (0.85–1.24 in men and 0.70–1.03 in women) | 3.01(1.2-7.53) | 0..020 |  |
| Quartile 3 (1.25–1.86 in men and 1.04–1.50 in women) | 1.58(0.37-6.8) | 0.537 |  |
| Quartile 4 (≥1.87 in men and ≥1.51 in women) | 1(0.3-3.3) | 0.999 |  |
| Total cholesterol (mmol/L) |  |  | 0.068 |
| Quartile 1 (<4.34 in men and <4.40 in women) | 1.69(0.66-4.29) | 0.27 |  |
| Quartile 2 (4.34–4.99 in men and 4.40–5.04 in women) | 3.83(1.45-10.14) | 0.008 |  |
| Quartile 3 (5.00–5.69 in men and 5.05–5.74 in women) | 0.3(0.05-1.81) | 0.185 |  |
| Quartile 4 (≥5.70 in men and ≥5.75 in women) | 1.19(0.24-5.83) | 0.826 |  |
| HDL-cholesterol (mmol/L) |  |  | 0.451 |
| Quartile 1 (<0.98 in men and <1.22 in women) | 1.45(0.51-4.09) | 0.482 |  |
| Quartile 2 (0.98–1.16 in men and 1.22–1.45 in women) | 2.59(0.87-7.69) | 0.085 |  |
| Quartile 3 (1.17–1.40 in men and 1.46–1.73 in women) | 0.9(0.15-5.25) | 0.902 |  |
| Quartile 4 (≥1.41 in men and ≥1.74 in women) | 3.31(0.7-15.72) | 0.131 |  |

All data were adjusted for age, gender, race, education level, income, cigarette smoking, alcohol use, drug addiction, BMI and abnormal medical conditions including gum problem, hypertension, diabetes.

**Supplementary Figure 1. The mediation Analysis of gum problems between denture and cardiovascular diseases**


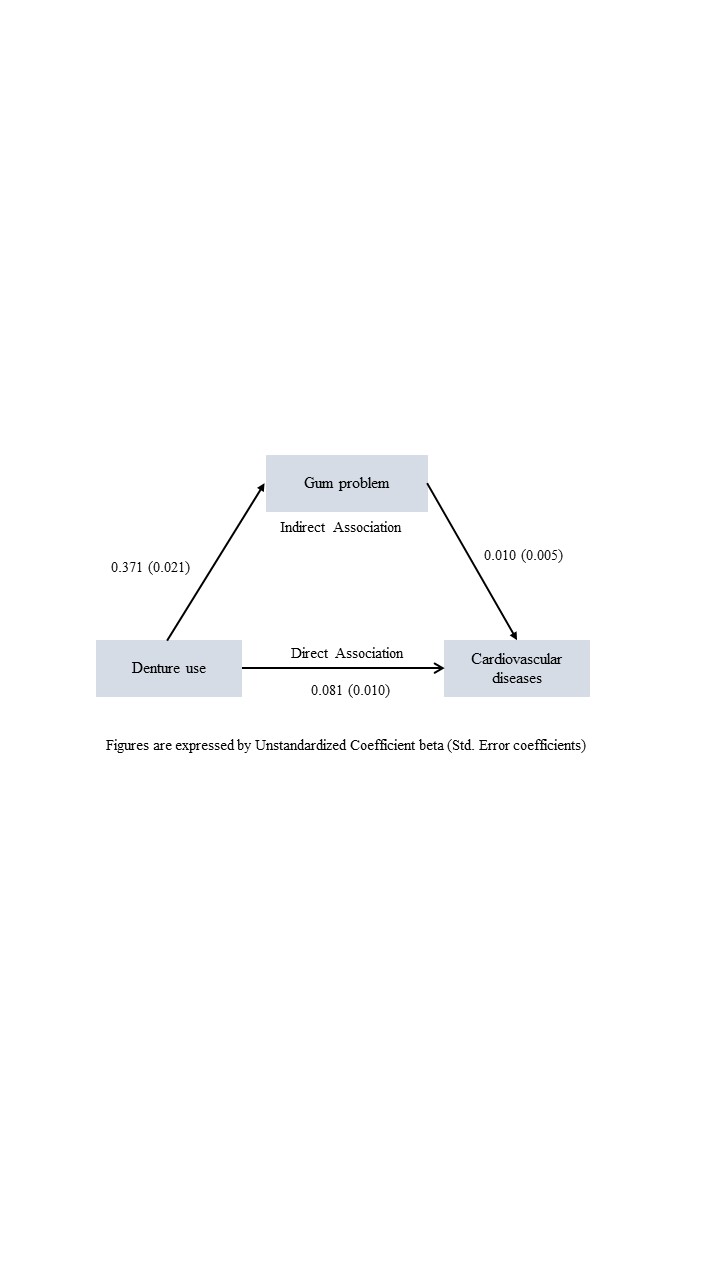


The indirect effect analysis result for denture use – gum problem – cardiovascular diseases using Sobel Test as follows:

Test statistic= 1.99

Std. Error=0.002

P-value =0.06 > 0.05
